# Mouse Model of Respiratory Tract Infection Induced by *Waddlia chondrophila*

**DOI:** 10.1371/journal.pone.0150909

**Published:** 2016-03-07

**Authors:** Ludovic Pilloux, Didier LeRoy, Christophe Brunel, Thierry Roger, Gilbert Greub

**Affiliations:** 1 Center for Research on Intracellular Bacteria (CRIB), Institute of Microbiology, University Hospital Center and University of Lausanne, Lausanne, Switzerland; 2 Infectious Diseases Service, Department of Medicine, University Hospital Center and University of Lausanne, Lausanne, Switzerland; 3 Institute of Pathology, University Hospital Center and University of Lausanne, Lausanne, Switzerland; University Freiburg, GERMANY

## Abstract

*Waddlia chondrophila*, an obligate intracellular bacterium belonging to the *Chlamydiales order*, is considered as an emerging pathogen. Some clinical studies highlighted a possible role of *W*. *chondrophila* in bronchiolitis, pneumonia and miscarriage. This pathogenic potential is further supported by the ability of *W*. *chondrophila* to infect and replicate within human pneumocytes, macrophages and endometrial cells. Considering that *W*. *chondrophila* might be a causative agent of respiratory tract infection, we developed a mouse model of respiratory tract infection to get insight into the pathogenesis of *W*. *chondrophila*. Following intranasal inoculation of 2 x 10^8^ *W*. *chondrophila*, mice lost up to 40% of their body weight, and succumbed rapidly from infection with a death rate reaching 50% at day 4 post-inoculation. Bacterial loads, estimated by qPCR, increased from day 0 to day 3 post-infection and decreased thereafter in surviving mice. Bacterial growth was confirmed by detecting dividing bacteria using electron microscopy, and living bacteria were isolated from lungs 14 days post-infection. Immunohistochemistry and histopathology of infected lungs revealed the presence of bacteria associated with pneumonia characterized by an important multifocal inflammation. The high inflammatory score in the lungs was associated with the presence of pro-inflammatory cytokines in both serum and lungs at day 3 post-infection. This animal model supports the role of *W*. *chondrophila* as an agent of respiratory tract infection, and will help understanding the pathogenesis of this strict intracellular bacterium.

## Introduction

Pneumonia is the third deadliest infectious disease worldwide, responsible for more than 3 million deaths per year [1]. Whereas hospital acquired (nosocomial) pneumonia primarily results from *Pseudomonas aeruginosa* infection, community acquired (CAP) is mainly due to *Streptococcus pneumoniae* infection. Others bacterial pathogens such as *C*. *pneumoniae* [2], *Legionella pneumophila* [3], and *Haemophilus influenzae* [4] also lead to pneumonia. It is therefore essential to identify the causative agent to select an effective antibiotic therapy [5,6]. Unfortunately, despite the availability of standardized diagnostic tools, in at least 50% of the cases of pneumonia the aetiological agent remains unidentified [7–9]. In this situation, pneumonia is likely initiated either by unknown, not diagnosable, bacteria or by atypical and fastidious bacteria that cannot be detected using routine diagnostic methods. Among the atypical and fastidious organisms, some *Chlamydia*–related bacteria, such as *Parachlamydia acanthamoebae* and *Simkania negevensis*, have been previously associated with respiratory tract infections [10–14]. Recently, *Waddlia chondrophila* (*W*. *chondrophila*), another *Chlamydia*-related bacterium, has been associated to respiratory tract infections such as pneumonia [15] and bronchiolitis in children [16]. These first clinical hints suggest a potential role of *W*. *chondrophila* in respiratory tract infections, which remains to be investigated.

As all members of the *Chlamydiales* order, *W*. *chondrophila* exhibit an obligate intracellular biphasic developmental cycle with reticulate bodies (RBs) that are metabolically active within a replicative niche called inclusion, and elementary bodies (EBs) that are metabolically inactive [17]. *W*. *chondrophila* was initially isolated from two bovine aborted foetuses in America [18,19] and in Germany [20]. Following veterinary investigations, *W*. *chondrophila* has been associated with bovine abortion [21,22], and a likely role of *W*. *chondrophila* in human adverse pregnancy outcome was documented [23–26]. There is up to now no evidence for a sexual transmission of *W*. *chondrophila*. However, association between *W*. *chondrophila* seropositivity and contact with animals supports a zoonotic mode of transmission [23].

*W*. *chondrophila* has been detected in well water [27], and is able to grow within free-living amoebae. Amoebae are therefore a potential important reservoir for *W*. *chondrophila*, and we should consider the water network as a potential transmission mode [28]. As it was demonstrated for *Legionella pneumophila* [29], *W*. *chondrophila* might be carried by amoebae within aerosolized water droplets, and inhaled upon contact with infected individual, animal or environmental reservoir. Moreover, *W*. *chondrophila* infect and grow very efficiently within a large panel of eukaryotic cells ranging from epithelial cells [30] to immune cells [31,32]. Altogether, these observations suggest a possible role of *W*. *chondrophila* in human and animal respiratory tract infections. In this study, we developed a mouse model of respiratory tract infection induced by intranasal inoculation of *W*. *chondrophila* and describe the innate immune response to infection. Overall, the results support a pathogenic potential of *W*. *chondrophila* in mice and suggest a role of the bacteria in lung infections. In addition, this model might also be useful to elaborate therapeutic strategies directed against *W*. *chondrophila* infection and to further study the biology of *Chlamydia*-related bacteria.

## Material and Methods

### Bacterial strain and inoculum preparation

*W*. *chondrophila* ATCC VR-1470 were grown at 32°C within *Acanthamoeba castellani* as previously described [10]. After 5 days, the co-culture was harvested by centrifugation at 8500g during 10 minutes. Pellet was diluted in 2 mL of PBS and filtered through a 5 μm filter to further disrupt and eliminate amoebal cells and fragments. Bacteria were concentrated by centrifugation at 8500g during 10 min, and pellet was resuspended in 1 mL of PBS. Finally, bacterial load was indirectly quantified using a specific quantitative real-time PCR [16] targeting the 16S rRNA encoding genes, which are present in two copies in the genome of *W*. *chondrophila* [33]. Mock control was amoebae not infected with *W*. *chondrophila* but was subjected to the same procedure as the infected sample.

### Mouse model of infection

Eight to 12-week-old female C57BL/6 mice were obtained from Charles River Laboratories (L’arbesle, France) and acclimatized for at least 1 week before experimentation. All animal procedures were approved by the Office Vétérinaire du Canton de Vaud, Lausanne, Switzerland (authorization n° 876.8, 877.8, and 1860.1), and performed according to our institutional guidelines for animal experiments. In agreement with our state ethic veterinary committee, no analgesic was provided to animals, considering that these agents interfere with innate immune responses. For each mouse we attributed a severity score graded from 1 (ruffled fur) to 5 (death), with grade 4 attributed to moribund animals (*i*.*e*. animals with deficient mobility compromising access to food and water). Intermediates grades 2 and 3 are associated to ruffled fur and respectively one or two others characteristics among conjunctivitis, diarrhea or motility troubles. Animals were monitored at least once daily, and whenever a mouse reached a severity score of 1, all animals were then monitored every 8 hours until the end of the experiments. Moreover, mice were euthanized when they met a severity score of 4. There was no unexpected death during the entire set of experiments.

Practically, mice were anesthetized by inhalation of 3% isoflurane (Attane, Bayer Health Care, Monheim, Germany). Mice were held in vertical position, and a drop of 20 μL containing *W*. *chondrophila*, or the mock control, was deposited in mice snares. One minute after inoculation, mice regained consciousness. Body weight and general state of health was monitored once a day. Mice were sacrificed by CO_2_ inhalation at different times post-inoculation. Whole blood was taken to measure cytokine concentrations. Lungs were harvested and divided in three parts. Lungs were cut longitudinally in equal parts. The outer part was dedicated to histology/immunohistochemistry analyses. The inner part was then cut transversally in two equal parts, the upper for bacterial DNA quantification, and the lower for cytokine measurement.

### Bacterial DNA quantification in the lung samples

To have a similar amount of lung sample tested by PCR for the presence of *W*. *chondrophila*, DNA extraction was always done on the same part of each lung, the other parts being used for histology, immunohistochemistry and cytokines measurement. Briefly, waddlial DNA was extracted using the Wizard Genomic DNA purification kit (Promega, Madison, WI, USA) according to the manufacturer’s instructions, and quantified by quantitative real-time PCR assay as described previously [16]. Briefly, the reaction was performed using iTaq supermix with ROX (Bio-Rad, Reinach, Switzerland), 200 nM of forward primer (WadF4, 5′- GGCCCTTGGGTCGTAAAGTTCT-3′), 200 nM of reverse primer (WadR4, 5′CGGAGTTAGCCGGTGCTTCT-3′), and 100 nM of probe (WadS2, 5′-FAM-CATGGGAACAAGAGAAGGATG- BHQ-3′). Amplification and detection of PCR products were performed with the ABI Prism 7000 Sequence Detection System (Applied Biosystems, Rotkreuz, Switzerland) using the following cycling conditions: 2 min at 50°C, 3 min at 95°C followed by 45 cycles of 15 s at 95°C and 1 min at 60°C.

### Histopathology and Immunohistochemistry

Each lung was sagitally cut into two equal parts. The outer part of each lung was fixed for 48 hours at room temperature in neutralized 4% formaldehyde and then conserved at 4°C in PBS until paraffin embedding. Paraffin embedded lung sections were haematoxylin/eosin stained to monitor the degree of inflammation. Adjacent sections were also investigated for the presence of (i) *W*. *chondrophila*, using a polyclonal rabbit anti-*Waddlia* antibody, as described previously [22] and (ii) macrophages, using a monoclonal rat anti mouse F4/80 antibody (Catlag Medsystems, Buckingham, UK), according to manufacturer's instructions.

Slides were analysed under light microscopy by a pathologist. Briefly, inflammation was scored as a percentage of inflamed area compared to the total area of the lung samples. The presence of *W*. *chondrophila* was scored on a scale ranging from 0 to 4. 0 corresponds to absence of *W*. *chondrophila*, 1 to sporadic presence of cells containing *W*. *chondrophila*, 2 to a few scattered cells containing *W*. *chondrophila*, 3 to mean number of cells containing *W*. *chondrophila* without aggregate, and 4 to high number of cells containing *W*. *chondrophila* with presence of aggregates within these cells. Finally, the macrophage infiltration was scored on a scale ranging from 0 to 3. 0 corresponds to the presence of a few macrophages as in normal lungs, 1 to a weak infiltration of macrophages, 2 to a moderate infiltration of macrophages, and 3 to a heavy infiltration of macrophages.

### Cytokines measurement

Whole blood samples were centrifuged to obtain plasma, and the inner and upper part of each lung was disrupted with a TissueLyser II (Qiagen, Hilden, Germany). The cytokine concentrations in plasma and lung homogenates were quantified by enzyme-linked immunosorbent assay (ELISA), as described by the manufacturer (R&D Systems, Minneapolis, MN, USA).

### Electron microscopy

Pieces of 2 millimetres diameter of lungs were harvested by using a biopsy punch device (Stiefel laboratories, Research Triangle Park, NC, USA) and fixed for 48 hours in 4% paraformaldehyde, 0.2% glutaraldehyde. Samples were washed in phosphate buffer and thin sections deposited on grids were analysed with a transmission electron microscope Philips EM 201 C (Philips, Eindhoven, The Netherlands).

### Co-culture assay

Lung samples were collected and mechanically disrupted with 2 ml Dulbecco's Modified Eagle Medium supplemented with sodium pyruvate and glutamine (GE Healthcare, Glattbrugg, Zurich, Switzerland) and 10% fetal bovine serum (Connectorate, Dietikon, Switzerland) in gentleMACS™ tubes using a gentleMACS™ Dissociator (Miltenyi Biotec, Bergisch Gladbach, Germany). Serial dilutions of the cell suspensions were transferred on a 24-well plate containing Vero cells. Plates were centrifuged at 1790g for 10 min and incubated at 37°C with 5% CO_2_ for 3 h to allow internalization of *W*. *chondrophila*. After 3 hours, cells were washed and incubated for 7 days in supplemented DMEM containing 50 μg/ml of gentamycin (Bioconcept, Allschwil, Switzerland) and 30 μg/ml of ampicillin (Sigma-Aldrich, Buchs, Switzerland) were added to inhibit growth of contaminants microorganisms. Viability of *W*. *chondrophila* was assessed by using a specific qPCR on Vero cells 3 h and 7 days post-inoculation.

### Statistical analysis

Statistical analyses were processed using GraphPad Prism 6.05 software for Windows (GrahPad Software Inc., La Jolla California, USA). Survival curves were created using the Kaplan-Meier method. Analysis of variance followed by unpaired two-tailed Student's *t*-test was used to analyse cytokines concentrations, and statistical significance was depicted as * p value <0.01, **p value <0.001, and ***p value <0.0001.

## Results

In a preliminary experiment, using a wide log range of bacterial inoculum (from 1 x 10^6^ to 1 x 10^9^), we roughly estimated the lethal dose 50 (LD50) close to 1 x 10^8^ bacteria.

**Fig 1:**
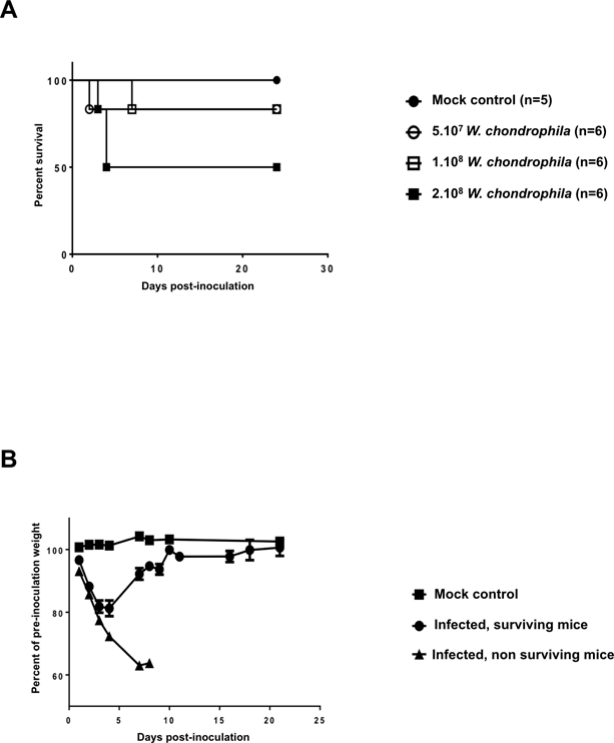
*Waddlia chondrophila* induce mice mortality and body weight loss. **A)** C57BL/6 mice (n = 6 animals per group) were inoculated intranasally with 5 x 10^7^, 1 x 10^8^ and 2 x 10^8^ *W*. *chondrophila*. A mock control group was processed in parallel (n = 5 mice). The Kaplan-Meier survival curve is shown. **B)** Body weight of mice that survived or died after *W*. *chondrophila* infection, and mock control treated mice. Results are expressed as the mean percentage (± SEM) of animal weight prior to inoculation.

Then, to analyse more precisely the pathogenicity of *W*. *chondrophila in vivo*, groups of six mice were inoculated intranasally with increasing quantities of *W*. *chondrophila* (5 x 10^7^, 1 x 10^8^ and 2 x 10^8^ bacteria) and survival monitored daily up to day 24 (**Fig 1A**). All deaths occurred between day 1 and day 7: one in the groups injected with 5 x 10^7^ and 1 x 10^8^ bacteria, and 3 in the group injected with 2 x 10^8^ bacteria. Death rate did not increase upon inoculation of 4 x 10^8^ bacteria, and no mortality was observed in the mock treated group, suggesting that the lethal dose 50 (LD_50_) was close to 2 x 10^8^ *W*. *chondrophila* in that model. Going well along with mortality, mice showed a rapid degradation of their global health status as reflected by a 36% lost of their initial weight at day 7 post-infection (**Fig 1B**). In contrast, animals to survive had a transient weight loss reaching 20% at day 5, and regained their initial weight at day 10 post-infection.

**Fig 2:**
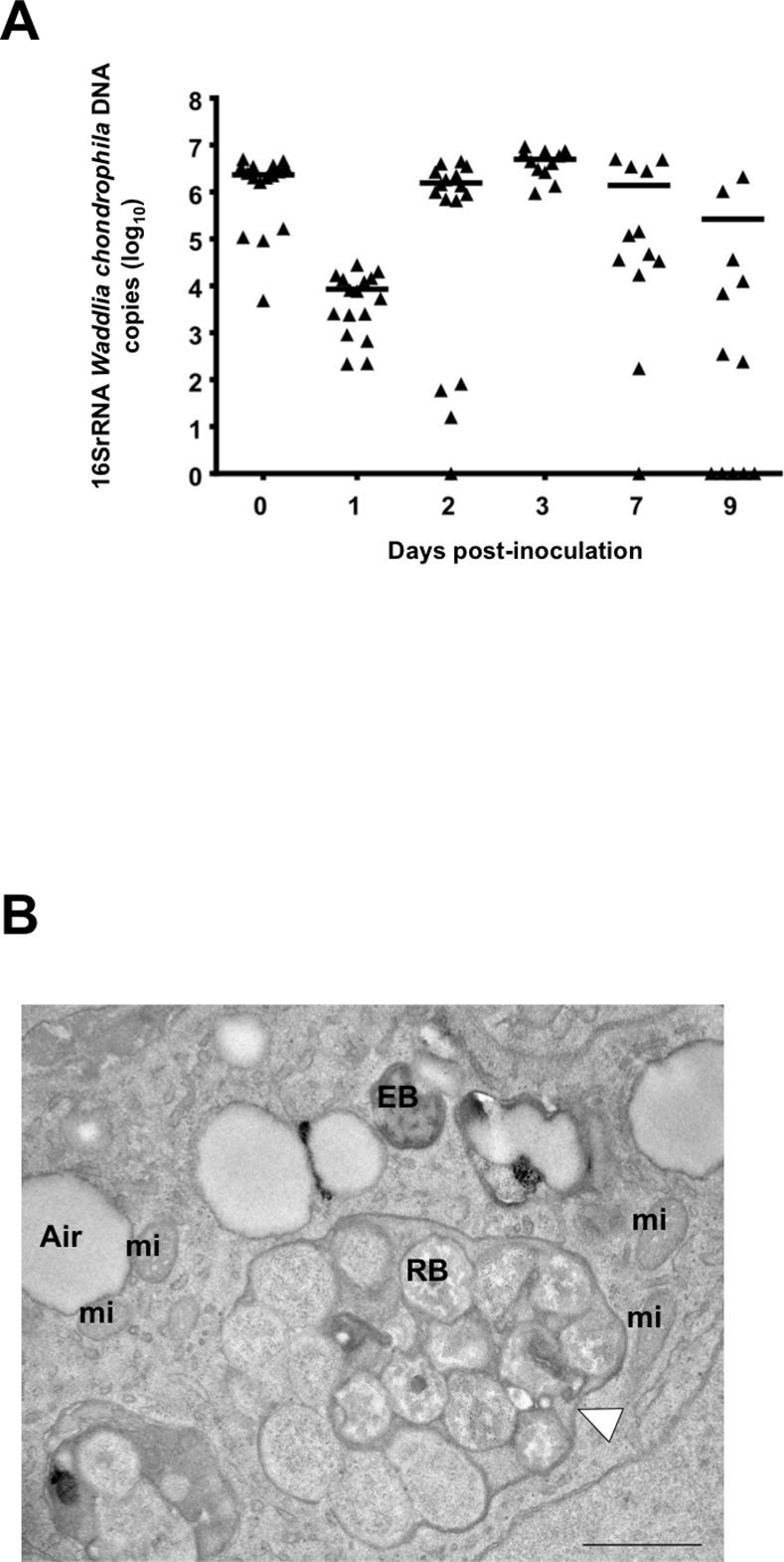
*Waddlia chondrophila* replicate within mouse lungs. **A)** Bacterial loads in lung homogenates of mice inoculated intranasally with 2 x 10^8^ *W*. *chondrophila*. The horizontal line represents the median value. **B)** Representative electron microscopy picture of a mouse lung 3 days post-inoculation with 2 x 10^8^ *W*. *chondrophila* (n = 3). The white arrowhead is showing an inclusion full of *W*. *chondrophila*. A: air in lung alveolus, EB: elementary body, RB: Reticulate body, mi: mitochondria. Note that *W*. *chondrophila* EB exhibits here a typical crescent shape that has been reported to be induced by fixative used for electron microscopy [41]. Scale bar = 1 μm. Magnification 13000x.

Bacterial loads in the lungs were assessed immediately and the following days after inoculation with 2 x 10^8^ *W*. *chondrophila* using a specific quantitative real-time PCR [16]. One hour after inoculation, 2.3 x 10^6^ (± 4 x 10^5^) bacteria were detected in lung samples (**Fig 2A**), indicating that approximately 1% of the inoculum reached the inner and lower part of the lungs 1 hour after inoculation. Bacterial loads dramatically dropped down during the next 24 hours, reaching 8.5 x 10^3^ (± 2 x 10^3^) bacteria per lung samples at day 1 post-inoculation. From day 1 to day 3, bacterial loads increased up to 6 x 10^6^ (± 7 x 10^5^) bacteria per lung samples. Bacterial loads then decreased down to 1.4 x 10^6^ (± 6 x 10^5^) at days 7, and 2.6 x 10^5^ (± 2 x 10^5^) at day 9, at which time points bacteria were not detected in 1/6 and 2/6 animals, respectively (**Fig 2A**). Bacterial burden at day 3 likely reflected sustained bacterial replication since dividing bacteria were regularly detected by electron microscopy analysis of lung sections collected 3 days post-infection (**Fig 2B**). Noteworthy, using a co-culture assay, living *W*. *chondrophila* were recovered from lungs up to 14 days post-infection. After lungs disruption, *W*. *chondrophila* were able to infect and grow within Vero cells proving they were alive. Moreover, low but reproducible amounts of bacterial DNA were amplified from the spleen and kidneys of mice infected with *W*. *chondrophila*, but not in lungs, spleen and kidneys of mock control animals (**data not shown**). Altogether, these data suggested that *W*. *chondrophila* was able to replicate efficiently within mouse lungs.

**Fig 3:**
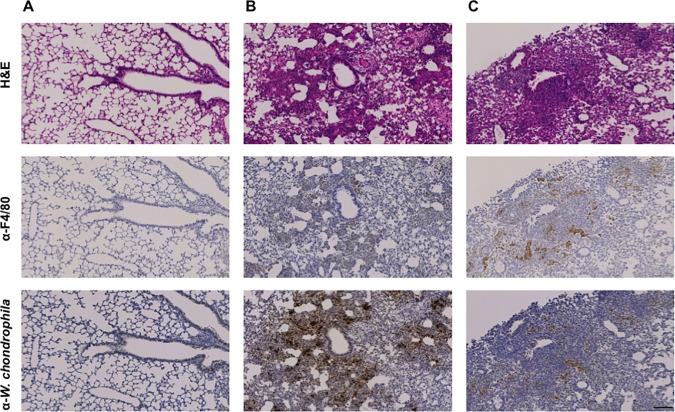
*Waddlia chondrophila* induce severe pneumonia. Mice were inoculated intranasally with 2 x 10^8^ *W*. *chondrophila*. Adjacent lung sections were stained with haematoxylin and eosin, anti-F4/80 antibody, and specific polyclonal anti-*W*. *chondrophila* antibody at different times post-inoculation. Scale bar = 100 μm. **A)** One hour post-inoculation, no signs of inflammation and no macrophage recruitment were observed. Only rare bacteria were detected within lung cells. Magnification 100x. **B)** Three days post-inoculation, we observed a subacute inflammation associated with a moderate recruitment of macrophages, and a massive infiltration of *W*. *chondrophila*. Magnification 100x. **C)** Ten days post-inoculation, we observed a moderate inflammation since the lympo-plasmacytic infiltration has slightly decrease, whereas there was still recruitment of macrophages in the rare areas where *W*. *chondrophila* was still persistent. Magnification 100x.

**Fig 4:**
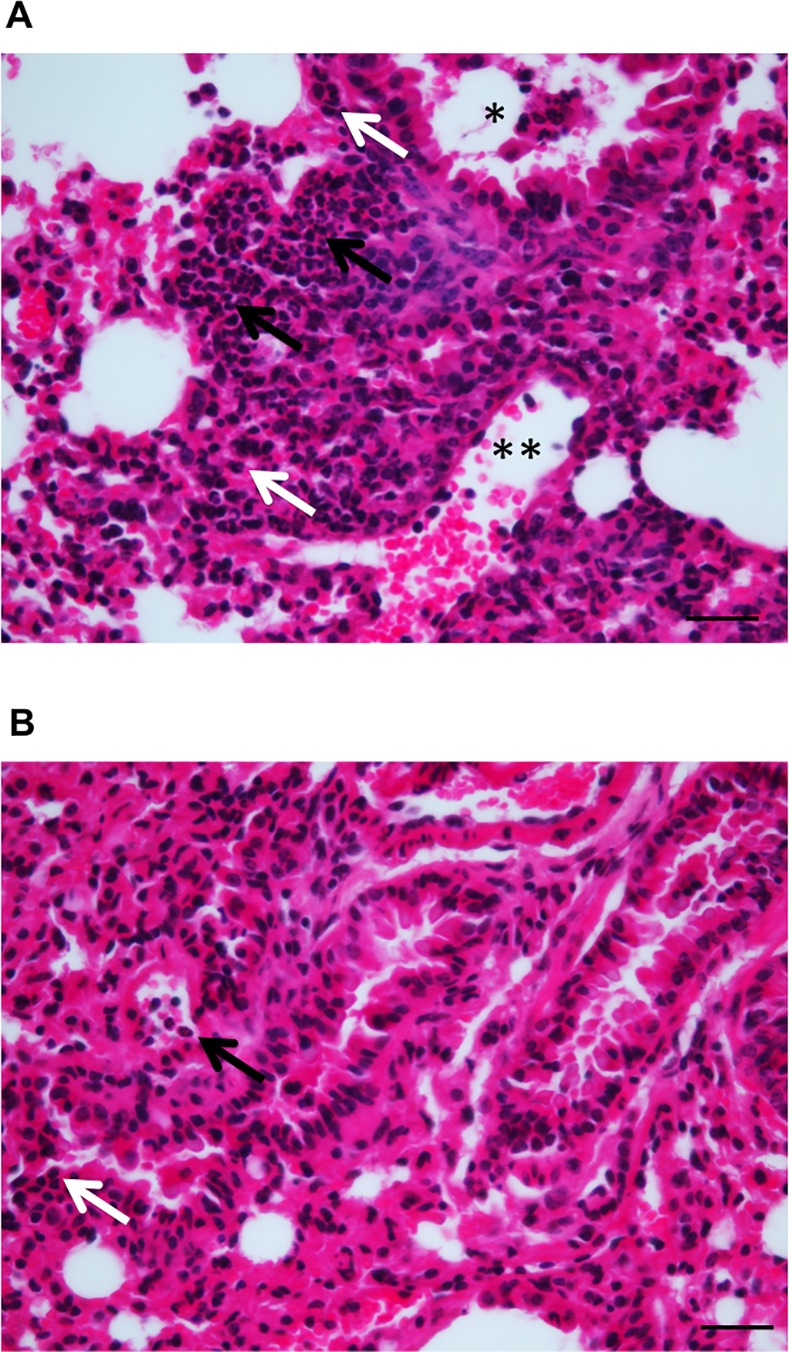
Dense infiltration of specific immune cells in the lungs of infected mice. Mice were inoculated intranasally with 2 x 10^8^ *W*. *chondrophila* or a mock control. Adjacent lung sections were stained with haematoxylin and eosin 3 days post-inoculation. Scale bar = 50 μm. **A)** Typical dense lympho-plasmacytic (white arrows) and neutrophilic (black arrows) infiltration of the lung alveolar and interstitial spaces of mice infected with *W*. *chondrophila*. Infiltration is peri-bronchiolar (black star) colonization of the bronchiole by neutrophils and lymphocytes that is typical of a subacute pneumonia. Magnification 400x. **B)** Typical focal inflammation in lungs of mock control mice, with presence of a few neutrophils (black arrow) and more lymphocytes (white arrow) in peri-bronchial and peri-bronchiolar area. Magnification 400x.

We then investigated the pathogenesis of *W*. *chondrophila*-induced lung infection by histology and immunohistochemistry methods (**Fig 3 and Table 1**). One hour post-infection with 2 x 10^8^ bacteria, only few infected cells were detected by immunohistochemistry using a polyclonal anti-*Waddlia* antibody. The proportion of infected cells increased from day 1 to day 3 at which a strong positive signal was recorded. At day 10 post-infection, only some positive areas were detected, suggesting that bacteria were either cleared from the lungs or disseminated. The transient increase of bacterial loads was associated with moderate macrophage recruitment at bacteria foci at day 3 and persistence of these macrophages up to day 10 post-infection. Signs of inflammation were detected early after inoculation, peaked at day 7 and then decreased with 34%, 44%, 57.5% and 33% of the lung area inflamed at day 1, 3, 7 and 10 post-infection, respectively (**Fig 3 and Table 1**). The severe inflammation was characterized by an important neutrophilic and lympho-plasmacytic infiltration of alveolar spaces and interstitial spaces (**Fig 4A**) with a predominantly peri-bronchial and peri-bronchiolar pattern that is characteristic of a subacute pneumonia. Of note, focal inflammation characterized by the presence of lympho-plasmacytic cells was observed in 30% of the lung surface area of mock treated animals at day 3 (**Fig 4B**).

**Table 1:** Detection of bacteria, inflamed area and macrophage infiltration in the lungs of mice infected with *Waddlia chondrophila*.

Day (n)	0 (8)	1 (8)	3 (6)	7 (6)	10 (6)
Presence of Waddlia chondrophila*	0.75	2.7	3.8	1.7	0.7
Inflamed area (%)**	18	34	44	57.5	33
Macrophage infiltration***	0	0	1.6	1.8	1

* The detection of *W*. *chondrophila* within cells was scored on a scale from 0 (no *Waddlia*) to 4 (strong presence of *Waddlia*).

** Inflamed area is expressed as the percentage of total lung area.

*** Macrophage infiltration was scored on a scale from 0 (no infiltration) to 3 (strong infiltration).

At day 3, neither macrophage infiltration and nor *W*. *chondrophila* were detected in mock controls (n = 6), whereas focal inflammation was observed in one third of the animals.

**Fig 5:**
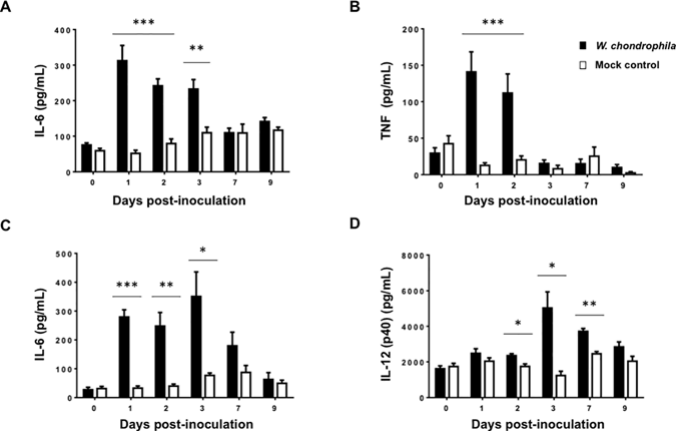
*Waddlia chondrophila* induce the secretion of pro-inflammatory cytokines in the blood and in the lungs of infected mice. IL-6, TNF and IL-12p40 in lung homogenates **(A and B)** and blood **(C and D)** of mice inoculated with 2 x 10^8^ *W*. *chondrophila* (black boxes) and mock controls (white boxes) were quantified by ELISA. Data are means ± SEM. n = 6–8 mice per group.

To gain insights into the inflammatory response induced by intranasal inoculation of *W*. *chondrophila*, we quantified pro-inflammatory cytokines in lung homogenates. When compared to mock controls, the concentrations of IL-6 were strongly increased at days 1, 2 and 3 following infection (**Fig 5A**), and that of TNF at days 1 and 2 (**Fig 5B**). Moreover, blood levels of IL-6 at days 1, 2 and 3 and IL-12p40 at days 2, 3 and 7 were significantly up regulated following infection with *W*. *chondrophila* (**Fig 5C and 5D**).

Altogether, these data confirmed the role of *W*. *chondrophila* as a respiratory pathogen responsible for severe lung inflammation, pneumonia and associated mortality in mice, further strengthening the potential pathogenic role of *W*. *chondrophila* in humans.

## Discussion

Clinical and experimental data suggest a possible role of *W*. *chondrophila* in respiratory tract infections [15,16,30]. It is thus essential to improve our understanding of the pathogenesis of infections induced by *W*. *chondrophila*. In that perspective, we established a murine model of respiratory tract infection induced by intranasal inoculation of *W*. *chondrophila*.

Bacteria were inoculated intranasally because this route might represents a natural way of infection, and because this simple procedure is less invasive for the animals than other approaches [34]. Half of the mice succumbed 3 days post-inoculation with 2 x 10^8^ bacteria, and animals going to die lost close to 40% of their initial weight. The inoculum of *W*. *chondrophila* used in this study was approximately ten times higher than inocula usually used in models of pneumonia induced by *C*. *pneumoniae* [35,36]. This inoculum corresponds to the lethal dose 50 (LD50) useful to describe the role of *W*. *chondrophila* in respiratory tract infections but clearly higher than the inocula that we expect to observe in naturally-occurring community-acquired pneumonia. Moreover, *Parachlamydia acanthamoebae*, another *Chlamydia*-related bacterium induced a clinical picture similar to that observed using *W*. *chondrophila* with nearly the same inoculum [12].

Interestingly, one hour after inoculation with *W*. *chondrophila*, we could detect about 2 x 10^6^ bacteria per lung samples corresponding to only 1% of the inoculum deposited into mice snares. The remaining bacteria could be still in the upper respiratory tract, exhaled, or be deviated into the digestive tract. One of the limitations of the intranasal inoculation technique is the variability of lung deposition of the microorganism among different mice [34]. Yet, whereas bacterial loads detected in the lungs early after inoculation were indeed heterogeneous ranging from 5.7 x 10^4^ to 2 x 10^6^ bacteria per lung, 24 hours later around 1 x 10^4^ bacteria per lung were measured in nearly all infected mice, demonstrating the robustness of our model. The early decrease of bacterial loads observed during the first day could be due to bacterial exhalation and dissemination into the bloodstream. Contrary to what was reported in mouse models of infection with other *Chlamydiales* [12,35,36], bacterial loads burst between day 1 to day 3 post-infection, reaching 6 x 10^6^ bacteria per lung samples. This increase probably resulted from a huge replication of *W*. *chondrophila*, as suggested by electron microscopy analyses showing dividing reticulate bodies in lung sections. The detection of bacterial DNA in spleen and kidney could represent some bacterial DNA disseminating from the initial site of infection through bloodstream, or could indicate that the *W*. *chondrophila* bacteria disseminated from the primary site of inoculation. This dissemination, associated with a huge local replication, might be responsible of the rapid fatal outcome of infection. Noteworthy, considering the possible role of *W*. *chondrophila* in bovine abortion and in adverse pregnancy outcomes in humans, advanced investigations might be useful to investigate for a potential tropism of *W*. *chondrophila* for genital tract organs.

Reminiscent of observations obtained following intranasal infection with *Parachlamydia acanthamoebae* [12], *W*. *chondrophila* bacterial loads decreased over time in surviving animals, reaching about 2.65 x 10^5^ bacteria per lung at day 9. Most likely, bacteria were eliminated by resident and infiltrating innate immune cells, as progressive clearance was associated with acute and subacute lung inflammation characterized by dense neutrophils and lympho-plasmocytes infiltration of the alveolar spaces at day 3 and day 7, respectively. Macrophages infiltration peaked at days 3–7 and decreased at day 10, which contrast with what was observed for *C*. *pneumoniae* infection [37]. Moreover, we did not observe signs of organizing pneumonia or interstitial fibrosis, in spite of the presence of a few remaining bacteria in the lungs. Further investigations should investigate for a potential long term persistence of bacteria, especially when considering the ability of *W*. *chondrophila* to differentiate into aberrant bodies, a non replicative form associated to persistent chlamydial infections [38,39]. Similarly to *C*. *pneumoniae*-induced pneumonia [40], *W*. *chondrophila*-induced pneumonia was associated with the production of pro-inflammatory cytokines such as IL-6 and TNF in the lungs, and IL-6 and IL12p40 in the blood. The observed acute/subacute inflammation was transient since 10 days after the onset of infection the pattern of inflammation was similar to that observed in the mock control group.

In conclusion, along previous observations suggesting a role of *W*. *chondrophila* as an emerging pathogen of respiratory tract infections [15,16], we successfully developed a reliable mouse model of pneumonia induced by intranasal inoculation of *W*. *chondrophila*. To complete the Koch’s postulate by establishing a link between a pathogen and a disease, *Waddlia* should be isolated from a patient suffering from pneumonia and grown in pure culture. The mouse model reported will help to increase our knowledge about *Chlamydia*-related pathogenesis, and will be a useful tool for future investigations including *in vivo* assessment of drug susceptibility and identification of virulence factors of *W*. *chondrophila*, as well as characterization of bacterial tropism and persistence.
